# Is Aberrant DNA Methylation a Key Factor in Molar Incisor Hypomineralization?

**DOI:** 10.3390/cimb44070197

**Published:** 2022-06-29

**Authors:** Wojciech Tynior, Danuta Ilczuk-Rypuła, Dorota Hudy, Joanna Katarzyna Strzelczyk

**Affiliations:** 1 Department of Medical and Molecular Biology, Faculty of Medical Sciences in Zabrze, Medical University of Silesia in Katowice, 40-055 Katowice, Poland; dorota@hudy.pl (D.H.); jstrzelczyk@sum.edu.pl (J.K.S.); 2 Department of Pediatric Dentistry, Faculty of Medical Sciences in Zabrze, Medical University of Silesia in Katowice, 40-055 Katowice, Poland; dilczuk-rypula@sum.edu.pl

**Keywords:** molar incisor hypomineralization, dental enamel hypoplasia, tooth abnormalities, DNA methylation, epigenomics, epigenetic mechanism

## Abstract

Molar incisor hypomineralization (MIH) is a qualitative disturbance of the enamel of the permanent molars and/or incisors. Its etiology is not clearly defined but is connected with different factors occurring before and after birth. It remains difficult to identify a single factor or group of factors, and the problem is further complicated by various overlapping mechanisms. In this study, we attempted to determine whether DNA methylation—an epigenetic mechanism—plays a key role in the etiology of MIH. We collected the epithelium of the oral mucosa from children with MIH and healthy individuals and analyzed its global DNA methylation level in each child using a 5-mC DNA ELISA kit after DNA isolation. There was no statistically significant difference between the global DNA methylation levels in the study and control groups. Then, we also analyzed the associations of the DNA methylation levels with different prenatal, perinatal, and postnatal factors, using appropriate statistical methods. Factors such as number of pregnancies, number of births, type of delivery, varicella infection (under 3 years old), and high fever (under 3 years old) were significantly important. This work can be seen as the first step towards further studies of the epigenetic background of the MIH etiology.

## 1. Introduction

Molar-incisor hypomineralization (MIH) is a disorder that causes a qualitative, congenital defect in permanent tooth enamel. Clinically, it manifests as white, yellow, or brown spot lesions on the surface of the permanent molars or/and incisors, visible during tooth eruption [1]. The pathogenesis of MIH, which has been defined as a complex condition, has been thoroughly investigated by a number of researchers [2,3,4]. Potential causal factors are divided into three groups: prenatal, perinatal, and postnatal. Prenatal factors include pathologies and maternal diseases during pregnancy, drugs, cigarette smoking, alcohol consumption, exposure to chemical and biological factors, ionizing radiation, and stress [3,5,6]. The group of perinatal conditions consists of time of delivery (weeks), type of delivery (natural labor/caesarean delivery), and complications during childbirth [4,6,7]. Postnatal factors that can be influential are fever episodes of more than 39 °C, breastfeeding, toxins (BPA), vitamin D_3_ deficiency, and infectious diseases (up to 3 years old): otitis, pneumonia, asthma, bronchitis, or varicella infection [2,5,6].

In recent years there has been an increased interest in exploring genetic factors in the development of MIH, and available studies indicate that specific genes such as *FAM83H*, *AMBN*, *BMP2*, *BMP7*, *BMP4*, *ENAM*, *MMP20*, *DLX3*, *FGFR1*, and *AMELX* may impact its development [8].

Experimental studies and many meta-analyses refer to numerous environmental and some genetic factors, but it remains difficult to indicate a single etiological factor or a group of factors with high statistical significance. Furthermore, the problem is exacerbated by different factors that impede the identification of the most relevant etiology mechanisms [9].

Understanding the etiology is important both from the diagnostic and therapeutic point of view as they help minimize the risk of MIH development and, if necessary, implement appropriate prevention and treatment.

We approach the problem with a broader perspective by focusing on epigenetic factors, molecular mechanisms that control gene expression without changing the sequences in DNA. One of these well-characterized mechanisms is DNA methylation, where methyl groups are added to cytosine in DNA by DNA methyltransferases [10,11]. Hypermethylated regions of DNA are associated with a reduced expression of genes, and consequently, the production of specific crucial proteins may be reduced. This mechanism is involved in the pathogenesis of different general disorders including cancers, autoimmune diseases, diabetes, hyperlipidemia, and neurodegenerative disorders such as schizophrenia and autism spectrum disorder [12,13,14]. Epigenetic modifications within the head and neck area contribute to periodontal disease, tooth development defects, and oral malformations [15].

Intensive molecular research, including epigenetic mechanisms, have helped to understand the etiology of different diseases for many years. DNA methylation is one of the major components of a cell’s epigenetic program. One of the ways of assessing DNA methylation is to measure the global level of DNA methylation as the percentage of 5-methylcytosine (5-mC) in the genome.

The aim of this study was to analyze the global DNA methylation levels among children with MIH and a control group. Afterwards, we also analyzed the associations of the DNA methylation levels with different potential environmental factors—prenatal, perinatal, and postnatal—recorded on a questionnaire.

The research is pioneering. The obtained results try to explain the role of DNA methylation in the development of MIH. Moreover, they fill the gap in the knowledge on the pathomechanism of MIH and contribute to the global understanding of this rare disorder. The presented results may become a stimulus for future, broader research.

## 2. Materials and Methods

### 2.1. Study Group and Sample Collection

Before dental appointments, two dental practitioners familiarized themselves with the European Academy of Paediatric Dentistry (EAPD) criteria [1] and the protocol of the research with exclusion/inclusion criteria (Table 1). They conducted physical examinations in line with checks of extended family medical history with a focus on the potential exposure to harmful environmental factors occurring before and after birth. Based on the dental examination, the children were classified into two groups: diagnosed with MIH—study group—and children without MIH—control group. Buccal epithelial scrapings were collected from children. The oral mucosa of cheeks was scraped with a cotton-swab stick and DNA was immediately isolated. All molecular analyses were performed at the Department of Medical and Molecular Biology, Faculty of Medical Sciences in Zabrze, Medical University of Silesia.

We examined 69 children from the Silesian Voivodeship (Poland). Two children were excluded because of craniomandibular and genetic disorders. The study included 67 children treated in the Developmental Age Dentistry Clinic in the Academic Centre of Dentistry and Specialized Medicine, Bytom, Poland. The study group consisted of 35 children aged 7–13 years diagnosed with MIH based on the criteria set out by the EAPD in 2003 (mean ± standard deviation (SD), 9.2 ± 1.4 years) and the control group consisted of 32 healthy children without any genetic disorders or malformations of dental tissues aged 8–12 years (mean ± SD, 9 ± 1.3 years). The study group consisted of 13 women and 22 men, whereas the control group had 17 women and 15 men. The population was homogeneous with respect to age (*p*-value = 0.31) and gender (*p*-value = 0.22).

### 2.2. DNA Isolation

DNA was extracted from buccal epithelial cells using a GeneMATRIX Swab-Extract DNA Purification Kit (EURx, Gdańsk, Poland) according to the manufacturer’s instructions. The quality and quantity parameters of the isolated DNA were analyzed using a spectrophotometer (NanoPhotometer Pearl, Implen, Munich, Germany).

### 2.3. Global DNA Methylation Analysis

There are various molecular methods to assess global methylation level. In our study, we utilized the enzyme-linked immunosorbent assay (ELISA)based kit. The global DNA methylation level was examined by a 5-mC DNA ELISA Kit (Zymo Research, Irvine, CA, USA, #D5325) as per the manufacturer’s protocol. Briefly, 100 nanograms (ng) of each DNA sample was used for analysis. The standard curve was generated using the absorbance values of 7 standards made by mixing the positive and negative controls supplied with the kit. The final methylation concentrations of standards were 0%, 5%, 10% 25%, 50%, 75%, and 100%, respectively. The percentage of 5-mC for unknown DNA samples was calculated using the equation:

% 5-mC= e{(Absorbance − y-intercept)/Slope}(1)

The absorbance was measured at 405 nm using an ELISA plate reader (BioTek Instruments, Inc., Winooski, VT, USA). All samples were analyzed in duplicates.

### 2.4. Statistical Methods

Results are presented as mean +/− SD. A Shapiro–Wilk test, Student’s *t*-test, U Mann–Whitney test and Fisher’s exact test were used to determine statistical significance at a *p*-value of <0.05. Correlations were analyzed with the Spearman method. All calculations were done in Statistica 13.1 and Microsoft Office Excel 2019.

## 3. Results

### 3.1. Global DNA Methylation in Study and Control Groups

**Figure 1:**
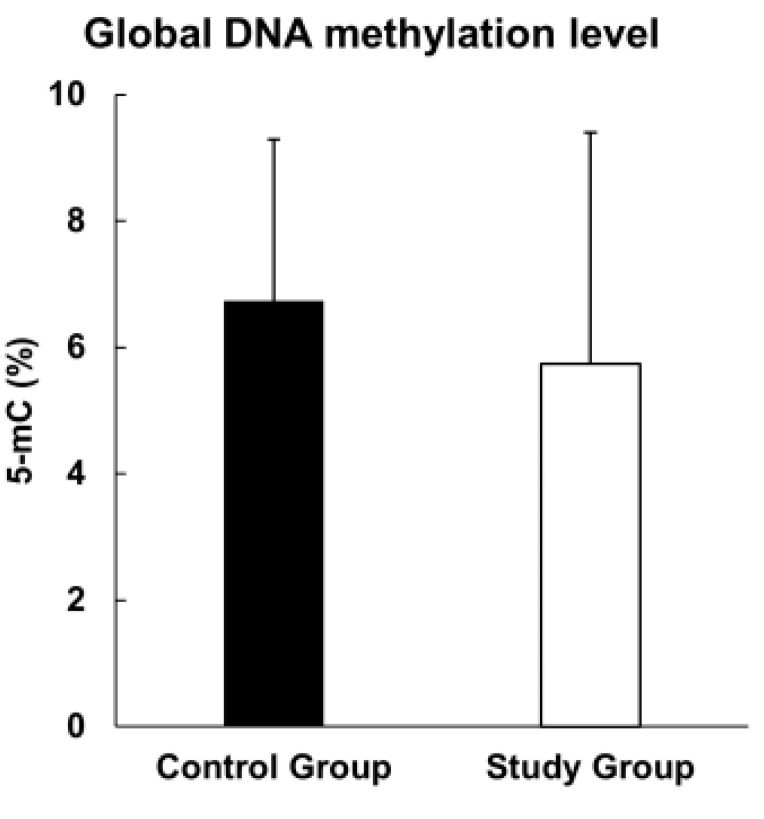
Global methylation level in study and control groups.

The mean global level of DNA methylation (as the concentration of 5-methylcytosine (5-mC) in genomic DNA) was different in the study group (5.73% 5-mC) and the control group (6.73% 5-mC), but this difference was not statistically significant (*p*-value = 0.209) (Figure 1).

Questionnaires related to 28 variables of possible etiological factors were filled out by parents of each participant to evaluate the association with global DNA methylation (Table 2).

### 3.2. Association between DNA Methylation Level and Potential Etiological Factors for MIH

**Figure 2:**
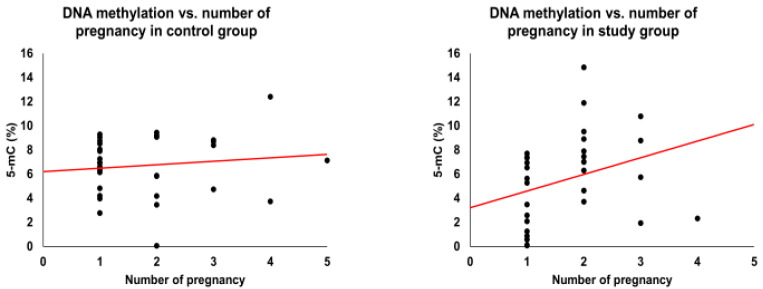
Global methylation level and number of pregnancies. The red line represents linear correlation.

**Figure 3:**
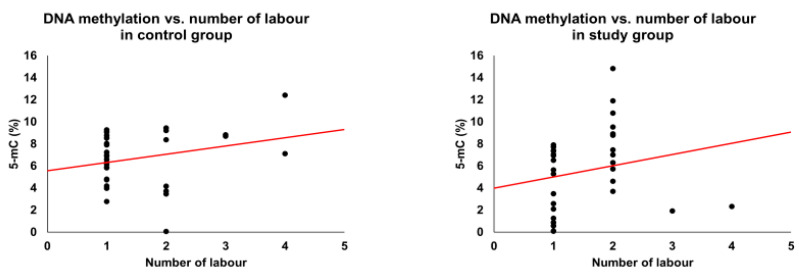
Global methylation level and number of labors. The red line represents linear correlation.

**Figure 4:**
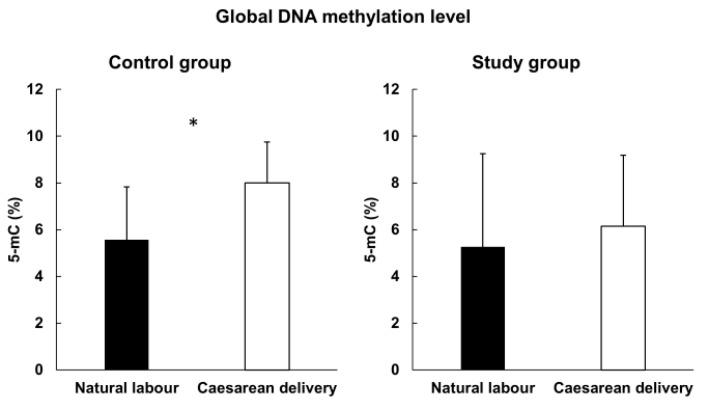
Global methylation level and type of delivery; *—statistically significant.

**Figure 5:**
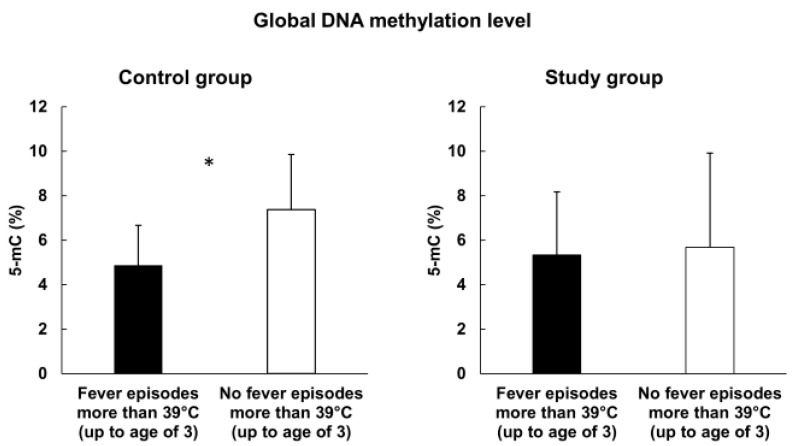
Global methylation level and fever episodes; *—statistically significant.

**Figure 6:**
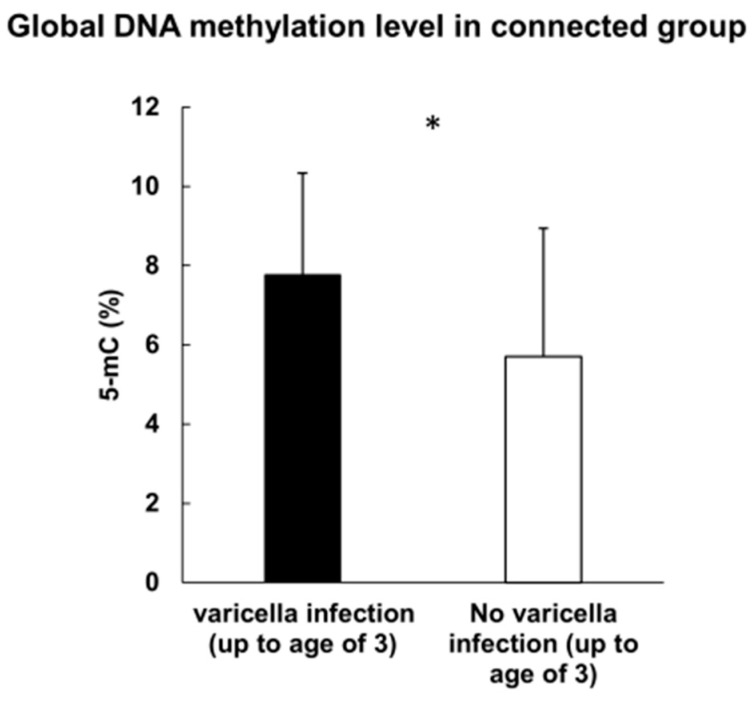
Global methylation level and varicella infection in the total group; *—statistically significant.

An association with global methylation level was seen for five of the factors in Table 1 (number of pregnancies, number of labors, type of delivery, varicella infection, and high fever (up to 3 years old) (Figure 2, Figure 3, Figure 4, Figure 5 and Figure 6). We observed in the study group that the number of pregnancies and the number of births were correlated positively (R^2^ = 0.52, *p*-value = 0.003) with the global methylation level: subsequent pregnancies and following children had a higher global methylation level than previous pregnancies and siblings (Figure 2 and Figure 3).

We showed in the control group, that children born via Caesarean delivery (CD) had a statistically significant higher global methylation level (mean 8.0% 5-mC) compared to natural-born children (mean 5.94% 5-mC) (*p*-value = 0.004) (Figure 4). Moreover, we observed that children who had not had high fever (under 3 years old) presented a higher global methylation level (mean 7.38% 5-mC) compared to those who had (mean 4.88% 5-mC) (*p*-value = 0.014) (Figure 5).

In the total group (MIH and control), children who had had a varicella infection (under 3 years old) presented a higher global methylation level (mean 7.77% 5-mC) compared to those who had not (mean 5.7% 5-mC) (*p*-value = 0.038) (Figure 6).

## 4. Discussion

Changes in DNA methylation can lead to the development of many pathologies. Abnormal DNA methylation status is involved in the pathogenesis of different disorders such as autoimmune diseases, cancers, diabetes, hyperlipidemia, and neurodegenerative disorders [12,13,14]. This wide range of different systemic diseases indicates a relevant participation of this epigenetic mechanism. We observe a growing interest in the field of epigenetics with a great effort devoted to assessing the etiology background of different diseases.

However, we found that the global DNA methylation level in buccal mucosa showed no statistically significant difference between children with MIH and healthy participants. On the other hand, lots of environmental factors such as drugs, smoking, alcohol consumption, and exposure to chemical and biological factors have an influence on MIH development, but the strict mechanisms remain unknown [16]. Moreover, Butera et al. claimed there was a high contribution of different genetic factors [2]. In the MIH field, great effort is devoted to the mechanical properties of the tooth tissues, enamel and dentin, and the most efficient treatment methods. According to our knowledge, there are no data regarding the contribution of epigenetic mechanisms in MIH development. After searching the database (PubMed and WoS) we did not find any research assessing global DNA methylation level in MIH patients. This is the first study to assess the global DNA methylation level in the group of MIH children.

We decided to collect buccal mucosa swabs, as the investigated material. The buccal mucosa is the most clinically accessible material and is valuable for the diagnostics of different oral diseases and conditions, especially among children. It has been confirmed by the number of genetic, molecular, and microbiological experiments carried out on oral diseases and the numerous studies on participants from the beginning of life to adolescence. Odintsova et al. used buccal swabs to show that breastfeeding was associated with epigenetic variation in buccal cells in children. A statistically significant effect was demonstrated in a group under 10 years old; four significant CpGs were associated with breastfeeding [17].

In this study, we established that among children diagnosed with MIH, who had older siblings, a higher level of global DNA methylation was observed in comparison with children who were born earlier, an outcome which might be linked with the age of the mother. Marshall et al. observed a higher level of global DNA methylation in oocytes of aged female mice in comparison to young females [18]. On the other hand, Markunas et al. identified a distinctive pattern of reduced DNA methylation in the blood of infants, which was correlated with the mother’s age at the time of labor [19], but they were not able to explain the mechanism through which the mother’s age impacts on the creation of permanent epigenetic alterations in offspring and suggested that these might be caused by inheritance of an altered maternal chromatin state. In a similar vein, Adkins et al. observed that in the subset loci, the level of DNA methylation in a child’s umbilical cord blood was strongly correlated with the mother’s age and to a lesser degree with the father’s [20]; this correlation was mostly negative and was disproportionately present in the area of CpG islands. However, Słabuszewska-Jóźwiak et al. concluded that there was no correlation between global DNA methylation in placentas and the number of parity [21].

We also established that children born through a Caesarean section presented a higher level of global DNA methylation in comparison with children born by natural birth. It is consistent with the results of Schlinzing et al. for the level of global DNA methylation from umbilical cord blood [22]. Słabuszewska-Jóźwiak et al. concluded that global DNA methylation was significantly lower in placenta from a planned Caesarean section in comparison to intrapartum Caesarean section and vaginal birth [21], although differences between intrapartum Caesarean section and vaginal birth were not statistically significant. Ducreux et al. made a genome-wide analysis of DNA methylation among children conceived naturally or via ART [23] and claimed that DNA methylation status was not significantly different in their cohort. Virani et al. concluded that the type of delivery of a newborn baby was not correlated with the level of global DNA methylation [24]. The significance of Virani’s research lies in both a large group as well as in the division of births of children and the use of two separate global methylation measurement techniques. Similarly, Franz et al. did not detect any differences in global methylation in newborn babies in groups of children born naturally in comparison with those born through a Caesarean section [25]. Moreover, Garot et al., based on a systematic review and meta-analysis, concluded that giving birth to a child through a Caesarean section may have an impact on the occurrence of MIH in the newborn [26].

Children who had not had a high fever (under 3 years old) presented a higher global methylation level compared to those who had had a fever. It is difficult to extract the effect of this factor alone because many infectious and noninfectious diseases also potentially influence DNA methylation. Fever is one of the most common symptoms in childhood [27] and refers to an infection or condition such as Behçet syndrome, Crohn’s disease, leukemia, or lymphoma. This difficulty is observed in urinary tract infections and pneumonia, which are the reasons of high fever episodes (higher than 40 °C) in children aged from 3 to 36 months of age [28]. However, we can refer to the different groups of research. There are studies concerning climatic conditions (including temperature) and their potential impact on DNA methylation. Xu’s study assessed the effect of ambient temperature on DNA methylation status, mostly in CpG islands and found that exposure to ambient temperature corresponded with DNA methylation in blood samples among Australian women from different regions [29]. The increase in average temperature was associated with the increase of DNA methylation in 31 CpGs. Bind et al. analyzed 777 elderly men—veterans from World War II and the Korean War—and claimed that temperature may reflect methylation of specific genes in blood cells [30]. They observed a 9% increase in DNA methylation in the ICAM-1 gene after a 5 °C increase during a three-week period. Nevertheless, available studies are short-term and limited to specific groups, and Xu et al. in a systematic review concluded that the evidence for an effect of environmental temperature remains insufficient [31].

In our total group (study plus control), children who had a varicella infection (under 3 years old) had a higher global methylation level compared to those who had not. The activity of the varicella-zoster virus (VZV) on host epigenome has not been proven. However, different viruses from the DNA virus family have broadly described the effect on the host’s DNA. Kuss-Duerkop et al. reviewed different groups of DNA tumor viruses and their implications for immune evasion and oncogenesis [32]. The viruses EBV, KSHV, HBV, HPV, SV40, MCPyV, and JC tend to block tumor suppressor genes. The study revised specific immune genes and groups of genes that are hypermethylated by viral activity and as a consequence deregulate host immune response. Strong evidence has been presented in HPV infection that has contributed to head and neck squamous cell carcinoma (HNSCC) development [33,34,35]. Epigenetic mechanisms can explain the overall cancer biology and pathogenesis. Moreover, epigenetic analyzes can be used as tools to diagnose and treat patients with HNSCC with high accuracy [34]. The group of onco-viruses remains well-characterized in the field of epigenetic contribution, whereas there is a lack of VZV virus studies and their potential epigenetic impact on the host genome.

A major strength of the study is the novel approach to the MIH etiology and noninvasive sample collection. We attempted to answer the question about the contribution of DNA methylation in MIH pathogenesis. The study did not reveal that the coincidence of MIH influenced the global DNA methylation status. However, similar environmental factors are responsible for both MIH development and DNA methylation alterations. We recommend new advanced methylation testing methods such as bisulfite sequencing or nanopore sequencing to validate our findings.

This study has some limits. It was constrained by the small quantity of participants in the research groups. We are planning to include more participants in the following studies.

Proving the association between DNA hypo-/hypermethylation of the specific genes, the occurrence of MIH, and the specific environmental factors will radically change molar-incisor hypomineralization etiology thesis. The research will fill the gap in the knowledge about the molecular mechanism of MIH, thus helping to minimize the risk of MIH development and, if necessary, implement appropriate prevention and treatment. We believe that it can be the first step towards further studies of the epigenetic background of the MIH etiology on a larger group.

## 5. Conclusions

Aberrant DNA methylation is an important epigenetic event in many diseases. Moreover, a lot of factors affect DNA methylation in different manners. Our study did not show a significant difference in global DNA methylation levels between children with MIH and healthy individuals. However, our observations confirm the presence of DNA methylation changes regarding environmental factors. Significant differences in global DNA methylation level were associated with 5 out of 28 potential prenatal, perinatal, and postnatal factors for MIH development (study group: number of pregnancies and number of births, control group: type of delivery and high fever (under 3 years old), total group: varicella infection (under 3 years old)). Considering the epidemiology of MIH and aspects of the interaction between genetic/epigenetics and environmental factors, we are planning more extended research.

## Figures and Tables

**Table 1:** Inclusion and exclusion criteria for the research.

Inclusion Criteria	Exclusion Criteria
Age 7–11 y.o.	Age <7 and >11
	Genetic disorders
	Birth defects
	Developmental defects of tooth (apart form MIH)

**Table 2:** Potential etiological factors for MIH development evaluated in the questionnaire.

	Variable
	Age of child MIH Gender
During pregnancy	Diseases Folic acid supplementation Vitamins supplementation Number of ultrasound examinations Drugs Alcohol Smoking cigarettes
During labor	Time for delivery (weeks) Type of delivery (natural labor/caesarean) Complications during childbirth Age of father at birth Age of mother at birth Number of pregnancies, Number of labors Body length (cm) Birth weight (g) APGAR scale Head circumference (cm)
Postnatal	Otitis (up to 3 years old), Pneumonia (up to 3 years old) Asthma (up to 3 years old), Bronchitis (up to 3 years old) Fever episodes more than 39 °C (up to 3 years old) Breastfeeding Varicella infection (up to 3 years old)

## Data Availability

Not applicable.
